# Mechanical Properties of High-Strength Cu–Cr–Zr Alloy Fabricated by Selective Laser Melting

**DOI:** 10.3390/ma13215028

**Published:** 2020-11-07

**Authors:** Fujia Sun, Ping Liu, Xiaohong Chen, Honglei Zhou, Pengfei Guan, Bilan Zhu

**Affiliations:** 1 School of Mechanical Engineering, University of Shanghai for Science and Technology, Shanghai 200093, China; liuping@usst.edu.cn (P.L.); 161330070@st.usst.edu.cn (X.C.); 183732500@st.usst.edu.cn (H.Z.); 172442512@st.usst.edu.cn (P.G.); 2 The Graduate School of Science and Engineering, Kagoshima University, Kagoshima 890-0065, Japan

**Keywords:** selective laser melting, laser power, scanning speed, hatching distance

## Abstract

The approximate process range for preparing the Cu–Cr–Zr alloy by selective laser melting (SLM) was determined by ANSYS simulation, and the influence of the SLM process parameters on the comprehensive properties of the SLM-formed alloy was studied by the design of experiments. The Cu–Cr–Zr alloy with optimum strength and hardness was prepared with high efficiency by optimizing the process parameters for SLM (i.e., laser power, scanning speed, and hatching distance). It is experimentally shown that tensile strength and hardness of the SLM alloy are increased by increasing laser power and decreasing scanning speed, whereas they are initially increased and then decreased by increasing the hatching distance. Moreover, strength, roughness and hardness of the SLM alloy are optimized when laser power is 460 W, scanning speed is 700 mm/s and hatching distance is 0.06 mm. The optimized properties of the SLM alloy are a tensile strength of 153.5 MPa, hardness of 119 HV, roughness of 31.384 μm and relative density of 91.62%.

## 1. Introduction

Due to their excellent thermal and electrical conductivity and mechanical properties, copper and its alloys are widely used in lead frames of integrated circuits, network cables for contacts in high-speed railways, nuclear-fusion heat-sink materials and aerospace heat-sink components [1]. Casting is the traditional production method for Cu–Cr–Zr alloys; however, it faces two problems: (i) difficulty in manufacturing copper alloy parts with complex structure and (ii) a long production cycle, which both make it difficult to meet the needs of the future. Copper alloy additive manufacturing (AM) uses computer-aided design (CAD) data, on the basis of the principle of layering and superposition of parts metal additive manufacturing technology. It can thus form complex structural parts when applied as rapid near net forming [2,3,4], including electron beam melting (EBM) [5,6], selective laser melting (SLM) [7,8], etc. EBM has been used for analyzing the influence of pure copper powder with variable bulk density on the properties of final formed materials, and copper samples with relative density up to 98.7% were prepared [9]. However, the surface quality of the samples formed by EBM is poor; therefore, SLM molding of copper and copper alloy has gradually started to receive attention [10,11].

Copper alloy has high thermal conductivity and low laser absorption, so it is difficult to prepare by SLM [12,13]. In recent years, with the development of optical-fiber laser technology, preparation of Cu–Sn [14], Cu–Al–Ni–Mn [15] and Cu–Ni–Si [16] alloys by SLM has progressed somewhat by increasing laser output energy and adding other elements to reduce thermal conductivity and improve absorptivity. For example, the Cu-Cr alloy with ultimate tensile strength of 468 MPa, yield strength of 377.33 MPa and conductivity of 98.31% IACS (International Annealed Copper Standard) was prepared with a 2000-W high power semiconductor laser by Zhang S. et al. [17]. Moreover, the Cu–Cr–Zr–Ti alloy with a density of 97.9% was prepared by SLM under certain conditions, i.e., laser power of 400 W, scanning speed of 400 mm/s and thickness of 50 μm, by Popovich A., et al. [18]. Highest tensile strength and elongation at room temperature perpendicular to the construction direction of the SLM-formed alloy can reach 211 MPa and 15.8%, respectively. The influence of different process parameters on properties of SLM-formed products has also been analyzed. For example, the influence of laser power, lap ratio, scanning speed and pulse frequency when forming material copper with a single cladding channel and a multiple cladding channel was analyzed by Pogson, et al. [19], and the influence of laser-energy density on the density and microstructure of tungsten copper was analyzed by Gu et al. [20]. Although the properties of copper alloy prepared by SLM have been preliminarily studied, the relationship between SLM process parameters and properties of the Cu–Cr–Zr alloy with 99.3% (units in wt %) copper content has not been reported. To understand the heat-transfer process of metal powder during melting, the influence of process parameters and material properties on SLM processing has been studied by numerical simulation. Two main methods (at the macro scale) for that simulation are available: (i) coupling of temperature and structure by using finite element analysis [21] and (ii) finite element analysis of inherent strain [22]. A three-dimensional thermal-fluid model using ANSYS/fluent was established by Subin et al. [23], who numerically analyzed temperature distribution, liquid–metal flow and free-surface formation in copper alloy parts. These studies mainly simulate the heat-transfer process of titanium alloy or alloy with low copper content. However, whether general commercial SLM equipment can meet the requirements of SLM processing of the Cu–Cr–Zr alloy has not been studied notably. Therefore, to improve the processing efficiency and quality of SLM equipment with an under-500-W laser, the influence of process parameters on the structure and properties of SLM-formed products should be studied.

In this study, accordingly, the appropriate laser power for SLM is obtained through absorption tests and ANSYS simulation. By comparing the microstructure and properties of the Cu–Cr–Zr alloy formed by using different SLM process parameters, the influence of laser power, scanning speed and hatching distance on the alloy properties was analyzed, and the optimum process parameters for attaining comprehensive SLM properties were obtained.

## 2. SLM Processes and Material Preparation

### 2.1. SLM Processes

Using a laser as an energy source, SLM and scans a bed layer of metal powder layer by layer according to a path planned by using a 3D CAD model. The scanned metal powder is combined metallurgically through melting and solidification in a manner that finally creates the metal part (SLM part, hereafter) designed by using the model. An metal-powder 3D printer (EP-M250, E-Plus-3D, Beijing, China) equipped with a 500-W Yb:YAG laser (with wavelength of 1064 nm and Gaussian spot diameter of 70 μm) was used to produce laser-powder-bed-fusion test specimens. The processing chamber was flooded with nitrogen gas to maintain the oxygen content below 100 ppm during the processing time.

The main parameters influencing the properties of SLM-formed alloys are laser power, scanning speed and hatching distance. To analyze the influence of these three parameters on the SLM alloy properties, it is first necessary to determine the required energy and approximate laser power. To do that, the laser absorptivity of Cu–Cr–Zr alloy powder was obtained by an integrating-sphere spectrometer test, and the power and energy required for melting the powder on the basis of that laser absorptivity were simulated by ANSYS simulation. The test and simulation are described in detail in Section 2.3 and Section 2.4. Sixteen sample blocks (with size of 25 mm × 25 mm × 6 mm) were printed by varying the three parameters (laser power, scanning speed and hatching distance), and the influence of each parameter on the SLM alloy properties was analyzed as described in Section 3.

### 2.2. Raw Alloy Powder

**Figure 1:**
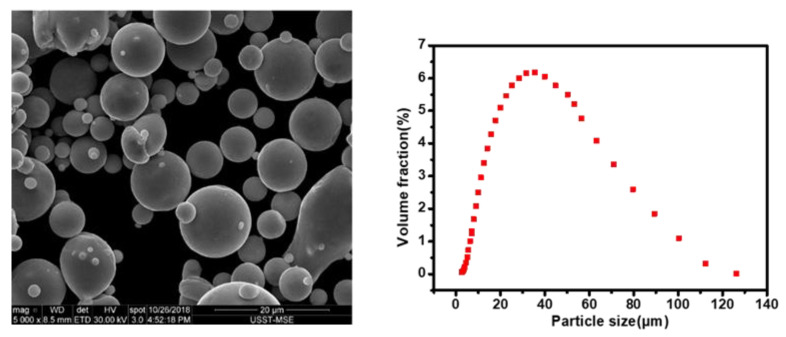
SEM micrograph and particle size distribution of raw Cu–Cr–Zr alloy powder.

Gas-atomized [24] (in argon atmosphere) Cu–Cr–Zr precipitation-strengthened alloy powder (−300 mesh, D50 of 30.268 μm, Supplied by Vilory, Xuzhou, China) with a spherical shape was selected as the starting material. The nominal chemical composition of the powder was 0.5–0.7% Cr, 0.06–0.15% Zr and a balance of Cu (units in wt %). Morphology, taken by a scanning electron microscope (SEM) and particle size distribution of the Cu–Cr–Zr alloy powder are shown in Figure 1. The particle-size distribution met the requirements for the SLM printer (EP-250) using alloy powder.

### 2.3. Laser Absorption Test of Cu–Cr–Zr Alloy Powder

When the laser irradiates the surface of the Cu alloy powder, part of the laser’s energy is reflected by the surface of the material, and the rest penetrates the powder. Part of the energy penetrating the powder is absorbed by the material. The absorptivity of laser irradiation on a Cu alloy surface can be calculated by using the Fresnel formula [25], given as:

(1) $$A=2\sqrt{2\omega^{\prime }\varepsilon_{0}/\sigma }=0.1457\sqrt{\rho /\lambda }$$

where, *A* is absorption rate, σ is electrical conductivity of the metal, *ω’* is frequency of the incident light, *ρ* is electrical resistivity of the metal material and *λ* is wavelength of the incident laser light. When the laser wavelength is 1064 nm, according to the formula, the absorption rate is only 0.0187.

**Figure 2:**
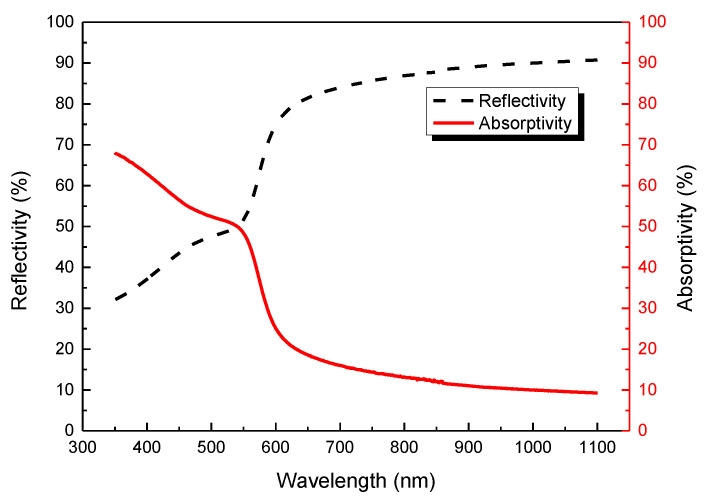
Absorption and reflectance curves of the Cu–Cr–Zr alloy tested at room temperature.

Shape of the formed SLM layer and placement of the powder determine the incidence angle of the laser, which affects the laser absorption rate. This is mainly due to the following two reasons: (i) multiple reflections of the laser on the powder surfaces are repeatedly absorbed and (ii) when the incidence angle is the Brewster angle, the laser absorption rate on the material surface is the largest, and almost all the beam energy can be absorbed. Therefore, a spectrometer was used in this experiment to measure the laser absorption rate at room temperature (as shown in Figure 2). According to the figure, when laser wavelength was less than 550 nm, laser absorption rate was higher however the wavelength used by the current SLM printer was 1064 nm, so the laser absorption rate was about 10.02%.

### 2.4. Prediction of Required Laser Power

The SLM laser printer uses a 500-W laser with a wavelength of 1064 nm and spot diameter of 70 μm. To obtain the printing parameters, APDL (ANSYS parametric design language) was used to establish the finite element model. Laser-beam intensity I in the z-direction can be expressed as:

(2) $$I\left(x^{\prime },y^{\prime },z^{\prime }\right)=\frac{2P^{\prime }}{\pi \omega^{2}\left(z^{\prime }\right)}\exp\left[-\frac{2\left({x^{\prime }}^{2}+{y^{\prime }}^{2}\right)}{\omega^{2}\left(z^{\prime }\right)}\right]$$

where *P’* is laser power, *ω(z’)* is spot radius at *z’* and light intensity is distributed according to a Gaussian function, $\exp\left[-\frac{2\left({x^{\prime }}^{2}+{y^{\prime }}^{2}\right)}{\omega^{2}\left(z\prime \right)}\right]$, in three directions (*x’*, *y’*, *z’*).

**Figure 3:**
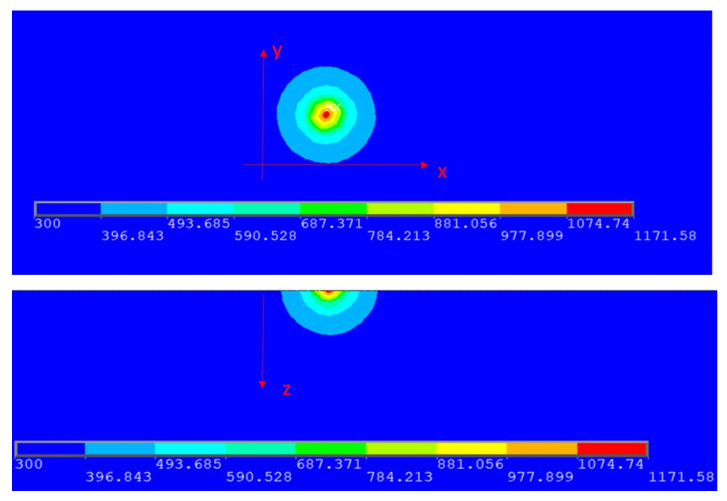
A temperature distribution of powder bed.

The temperature field in the laser-cladding process obeys the law of a nonlinear transient heat transfer. When the laser beam irradiates the defined area on the powder bed, a high gradient of temperature is generated between the molten pool formed by the laser and other parts of the matrix, and the heat is transferred from the laser to the molten matrix rapidly. The heat conduction equation is given as:

(3) $$\rho^{\prime }c\frac{\partial T}{\partial t}=\frac{\partial }{\partial x}\left(k\frac{\partial T}{\partial x}\right)+\frac{\partial }{\partial y}\left(k\frac{\partial T}{\partial y}\right)+\frac{\partial }{\partial z}\left(k\frac{\partial T}{\partial z}\right)+Q$$

where *ρ’* is the density of the copper alloy, which was taken as 88,900 kg/m^3^; *c* is the specific heat capacity of the copper alloy, which was taken as 390 J/kg·K; *T* is the distribution function of the temperature field, at time, *t* = 0, the workpiece has a uniform ambient temperature of 300 K; *t* is the heat-transfer time (in s), from *t* = 0 to *t* = 0.016 s; *k* is the thermal conductivity of the copper alloy, which is taken as 320 W/m·K; *Q* is the amount of heat absorbed (in J) and x, y and z indicate the three axial directions, respectively. *Q* in Equation (3) was calculated with Equation (1), *Q* = *P’* × *t* × *A*, and the results of an ANSYS simulation base on Equation (3) in two directions are obtained as shown in Figure 3 where *P’* (laser power) was taken as 459 W, *A* (laser absorption rate) was taken as 0.1002, and the numbers along the line at the bottom of the graphs show temperature.

As for the boundary conditions of the Cu–Cr–Zr alloy powder and laser, the simulation results show that when the laser was fully absorbed and laser power was about 46 W, the alloy powder could be heated to its melting point to form a molten pool after 0.16 ms. According to Figure 2, the actual powder absorption rate was about 10.02%; therefore, the required appropriate laser power was about 459 W (46 W = 459 W × 10.02%). The SLM printer used a 500-W laser, so it satisfied the heat requirement.

### 2.5. SLM Processes and Material Preparation and Characterization

The fabrication was carried out using the EP-250 SLM solution system (E-Plus 3D, Beijing, China) in an argon was performed atmosphere. Layers were formed using a continuous laser mode according to a cross pattern, and rotated 67 degrees between each layer. To analyze whether the Cr component affects the alloy, an XRD analysis. In particular, the phase structure of the raw powder and SLM alloy was characterized via X-ray diffraction (D8 Advanced, Bruker AXS, Karlsruhe, Germany). The density of the alloy fabricated under the different SLM process parameters was measured by the Archimedes drainage method. Relative density of the alloy was obtained by comparing measured density with theoretical density calculated.

(4) $$\rho_{\mathrm{relative}\;\mathrm{density}}=\frac{\rho_{\mathrm{actual}\;\mathrm{density}}}{\rho_{\mathrm{theoretical}\;\mathrm{density}}}$$

where $\rho_{\mathrm{relative}\;\mathrm{density}}$ is the relative density; $\rho_{\mathrm{actual}\;\mathrm{density}}$ of the copper alloy is measured by the Archimedes drainage method (in g/cm^3^) and $\rho_{\mathrm{theoretical}\;\mathrm{density}}$ is 8.89 g/cm^3^ of the Cu–Cr–Zr alloy.

**Figure 4:**
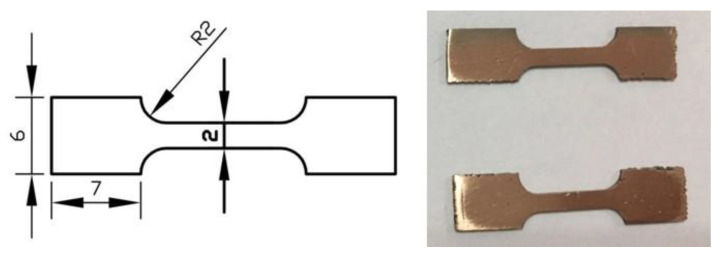
Tensile sample shape and size.

Vickers hardness (200-g load and 10-s loading time) of the SLM alloys was measured by using a universal hardness tester (HX-1000TM/LCD, Shanghai optical instrument factory, Shanghai, China) after the alloys were ground by sandpaper (with roughnesses of 400#, 800#, 1000#, 1500# and 2000#) and polished with diamond abrasive paste. At least ten impressions on the top surface of the samples were measured for each specimen to obtain a representative value. The standard deviation of the measurements was about 4 HV. Tensile strengths of different SLM alloy tensile specimens (cut by WEDM) were measured by using a microtensile testing machine (Zwick Precision Linevario, Zwickell, Ulm, Germany) at a room temperature with a loading rate of 2 mm/min. The values of tensile strength were recorded at the moment of the fracture, and the results derived from the average of three separate samples. Tensile test specimens (Figure 4) were sectioned from the SLM alloys, by using a water-cooled slow-feeding wire-cut machine in order to prevent unwanted thermal influence. The fracture morphology was observed by a scanning electron microscope (SEM) using Quanta FEG 450 (FEI, Hillsboro, OR, USA). Surface roughness of the SLM alloys was investigated by using confocal laser scanning microscope (OLS5000, Olympus, Shinjuku-ku, Tokyo, Japan).

## 3. Experiments and Discussion

### 3.1. Process Parameters

The main process parameters governing SLM are laser power, scanning speed, and hatching distance. To analyze the influence of these three parameters on the alloy properties, the 16 sample blocks (with size of 25 mm × 25 mm × 6 mm) were printed under selected process parameters as listed in Table 1. When each of the three parameters was given as four variables, the number of all combinations of the three parameters was 64. However, it is very time consuming to print the 64 sample blocks. The relationship between technological process parameters on the mechanical properties could be obtained by 16 representative fabricated sample blocks. By fixing two of the three influencing factors (printing power, scanning speed and hatching distance) in each group, these 16 representative sample blocks were selected for printing and evaluating the impact of the other on mechanical properties.

To ensure that other variables did not affect the experiment, the uniformity of layer thickness was set to 30 μm. In order to minimize the damage to the laser caused by reflection, 16 samples were evenly arranged around the powder bed. In this way, the influence of sample position on the relative density value can be ignored. To categorize the energy input for these parameter combinations, volume-based energy density was calculated as follows:

(5) $$E=\frac{P}{vNS}$$

where *E* represents volume energy density (in J/mm^3^); P is laser power (in W); v is scanning speed (in mm/s); N is hatching distance (in mm) and S is layer thickness (in mm). Energy density calculated from Equation (5) is shown in the last column in Table 1.

The 16 sample blocks were divided into four groups as shown in Table 1, where groups *A* and *D* were used to analyze how the alloy properties changed with changing scanning speed when hatching distance was constant, while groups *B* and *C* were used to analyze how the alloy properties changed with changing in hatching distance when scanning speed was constant.

### 3.2. XRD Analysis

**Figure 5:**
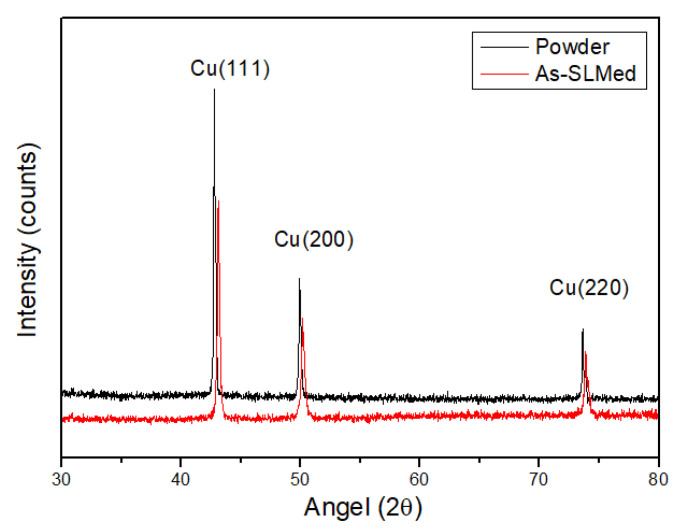
XRD patterns of the Cu–Cr–Zr raw alloy powder and as-built SLM alloy.

XRD spectra of the raw Cu–Cr–Zr alloy powder and SLM alloy are shown in Figure 5. It is clear from the figure that the alloy powder and SLM alloy exhibited the same phase structure as pure copper. They are composed of single-phase copper without the Cr phase because the content of Cr in the raw powder was only 0.5–0.7%, which was beyond the detection range of XRD for precipitated phase. After SLM forming, the diffraction peaks of the alloy shift to a larger angle. As for this result, the lattice constant of Cu in the SLM alloy was decreased compared to that in the raw powder for the following reason. The cooling rate of the alloy powder during SLM forming was high, so a substitutional solid solution was formed by a solid solution of Cr atoms in the lattice of Cu before it can precipitate. Since the Cr atoms were smaller than the Cu atoms, the intensity of the diffraction peak of the SLM alloy decreased and broadened. At the Cu(111) crystal plane, the intensity of the diffraction peak decreased significantly, and the half-peak width increased from 0.149 to 0.192, which indicates that partial recrystallization in the alloy might occur. The recrystallized grains could not grow, and the final crystallite size decreased from 62.4 to 41.3 nm because of the high cooling rate.

### 3.3. Effect of Process Parameters on the Relative Density of the SLM Alloy

**Figure 6:**
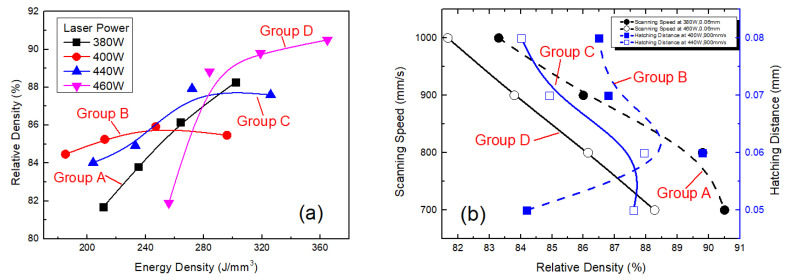
Relative density of the alloy formed by SLM under different process parameters: (**a**) relative density of the alloy versus energy density (laser power is labeled according to different groups via different symbols and legends) and (**b**) scanning speed and hatching distance versus the relative density of the alloy.

As relative density increased, fewer micron-sized holes formed inside the alloy, and the quality of the alloy improved. Change in relative density of the SLM alloy formed under varied laser power, scanning speed and hatching distance for groups *A, B, C* and *D* (where each line corresponds to a group) was plotted in Figure 6. Relative density of the alloy for groups *A, B, C* and *D* is shown in Figure 6a. As shown in the Figure 6a, the relative density of the alloy increased with increasing laser power at the same scanning speed and hatching distance. A high laser power provides a large amount of energy density, the alloy powder can be fully melted, and metallurgical bonding between the powder particles can be achieved, thereby increasing relative density. When the laser power was 460 W, scanning speed was less than 1000 mm/s, the hatching speed was kept constant at 0.06 mm and high relative densities (>90%) were achieved compared to other processing parameters used for other groups. In most cases, the influence of scanning speed on the density was greater than that of hatching distance when the power was the same. When the laser power was 400 W, scanning speed was 900 mm/s and relative density of the alloy was not obvious with the change of hatching distance. Effect of scanning speed on relative density for groups *A* and *D* and that of hatching distance on relative density for groups *B* and *C* are shown in Figure 6b. As shown in Figure 6b, relative density of the samples decreased with increasing scanning speed. As scanning speed was increased, the time the laser stayed on the powder surface decreased per unit time, the interaction time between the laser and powder became the shorter, and less heat was injected by the laser into the molten pool per unit time. As a result, maximum temperature and temperature gradient of the molten pool were lowered, and some of the alloy powder was not fully melted, resulting in many tiny voids between the powder particles. According to the figure, scanning speed of 700 mm/s brought the highest relative density. Note that a lower speed is impractical because the 3D printer would be damaged. Moreover, relative density of the samples initially increased and then decreased with increasing hatching distance. When the scanning interval was excessively large, some powder could not be metallurgically bonded, thereby reducing final relative density.

### 3.4. Effect of Process Parameters on the Hardness of SLM Alloy

**Figure 7:**
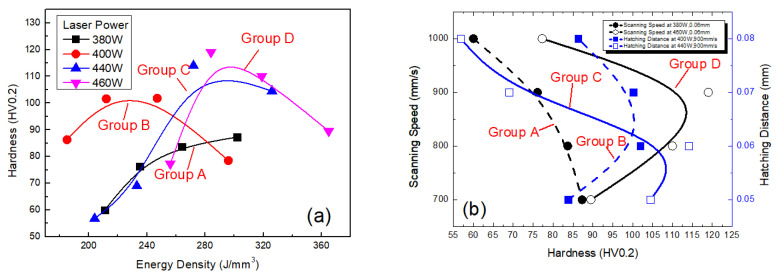
Hardness of the SLM alloy under different process parameters: (**a**) hardness of the alloy versus energy density (laser power is labeled according to different groups via different symbols and legends) and (**b**) scanning speed and hatching distances versus hardness of the alloy.

Variation of hardness of the SLM alloy with laser power, scanning speed and hatching distance for groups *A, B, C* and *D* (where a line corresponds to a group) is plotted in Figure 7. The results for groups *A, B, C* and *D* are shown in Figure 7a. As shown in the Figure 7a, hardness of the alloy was increased with increasing laser power at the same scanning speed and hatching distance. As laser power increased, output energy density was also increased, so strong metallurgical bonding between the powder particles was achieved. When pressure was applied to the SLM alloy, the strong metallurgical bonding between the particles hinders sliding of the grains over one another; therefore, hardness of the alloy was improved. Cu–Cr–Zr belongs to the precipitation hardened copper alloy [26,27,28]. When laser power was 460 W, scanning speed was 900 mm/s, the hatching speed was 0.06 mm and hardness of the SLM alloy rose to 119 HV, which might be due to the precipitation of some Cr atoms. Effect of scanning speed on hardness for groups *A* and *D* and that of hatching distance on hardness for groups *B* and *C* are shown in Figure 7b. As shown in the Figure 7b, hardness of the alloy decreased with increasing scanning speed for group *A*; however, this result shows group *D* as an exception. A high scanning speed results in low energy absorption per unit time, so the powder was not fully melted and then cooled, resulting in a large number of pores. It also resulted in low relative density. Hardness of the SLM alloy was thus also low. For group A, B and C, the scanning speed of 800 mm/s and the hatching distance of 0.06 mm was an inflection point. However, the result for group *D* shows a different variation, so it is considered an exception. For group D, the scanning speed of 900 mm/s and the hatching distance of 0.07 mm was an inflection point. The possible reason is that when scanning speed was larger than inflection point speed, and the hatching distance was greater than inflection point distance, Cr atoms were mainly precipitated, which improved the hardness; otherwise, Cr atoms were mainly solid solution, which reduced the hardness. For group *A*, when scanning speed was increased from 700 to 1000 mm/s, hardness of the SLM alloy decreased from 87.3 to 60 HV. Moreover, hardness decreased when hatching distance was excessively low or high. This result was consistent with the effect of hatching distance on relative density of the SLM alloy.

### 3.5. Effect of Process Parameters on Tensile Strength and Fracture Morphology of the SLM Alloy

**Figure 8:**
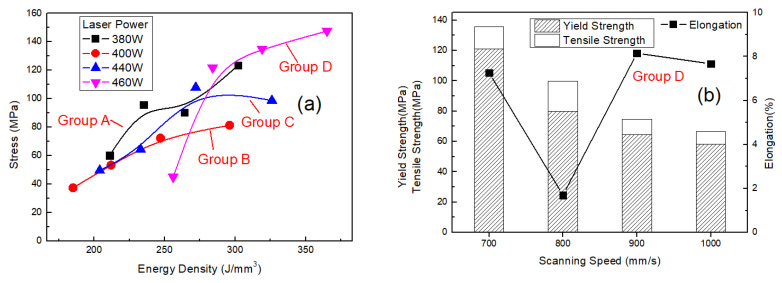

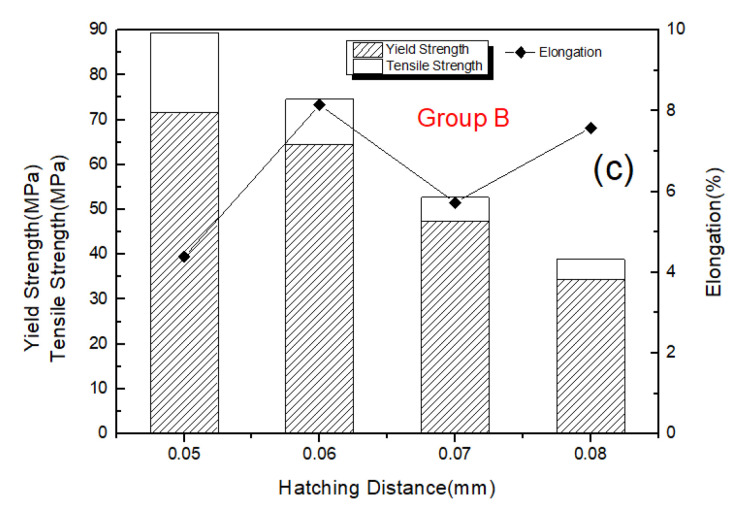
Strength curves of the SLM alloy formed under different process parameters: (**a**) strength of the alloy versus energy density (laser power is labeled according to different groups via different symbols and legends), (**b**) scanning speed versus strength of the alloy and (**c**) hatching distances versus the strength of the alloy.

**Figure 9:**
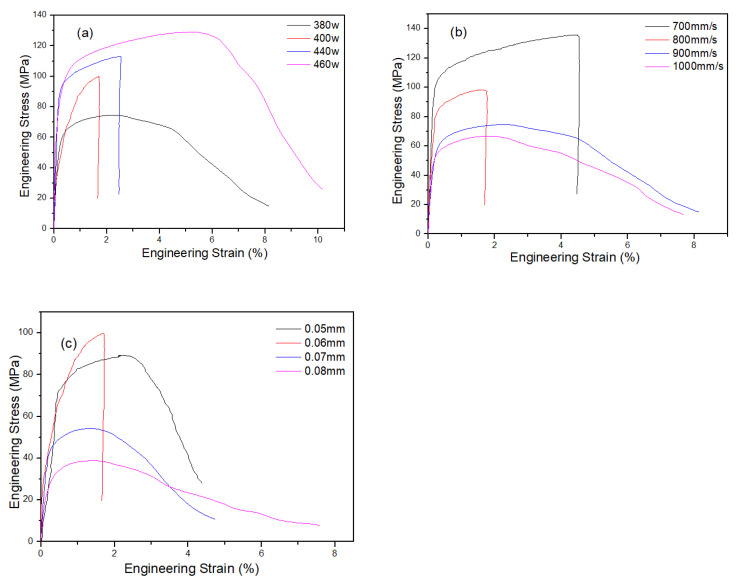
Stress–strain curves variation of the as-built samples with (**a**) laser power, (**b**) scanning speed and (**c**) hatching distance (in this case, (**a**) scanning speed of 900 mm/s, hatching distance of 0.06 mm; (**b**) laser power 380 W, hatching distance 0.06 mm and (**c**) laser power 400 W, scanning speed 900 mm/s).

The strengths of the SLM alloys fabricated under different process parameters also differed. Variations of strength curves and stress–strain curves of the as-built samples with laser power, scanning speed and hatching distance are plotted in Figure 8 and Figure 9. The results for groups *A, B, C* and *D* are shown in Figure 8a, which shows that tensile strength of the SLM alloy increased with increasing laser power. A high laser power resulted in strong metallurgical bonding of the SLM alloy and thus high tensile strength. Effect of scanning speed on tensile and yield strength for group *D* is shown in Figure 8b, which shows a tensile strength of the SLM alloy decreased with increasing scanning speed. High scanning speed resulted in low relative density and therefore low tensile strength of the SLM alloy. When scanning speed was 700 mm/s, tensile strength of the SLM alloy reached 135.7 MPa. Effect of hatching distance on tensile strength for group *B* is shown in Figure 8c, which shows that hatching distance of 0.05 mm gave the best result in terms of strength, and the strength for 0.06 mm was comparable to that for 0.05 mm. Moreover, tensile strength decreased with increasing hatching distance. To be consistent with the selection of hatching distance in relation to relative density and hardness of the SLM alloy, 0.06 mm could be selected as the optimum hatching distance for SLM.

### 3.6. Effect of Process Parameters on Fracture Morphologies of SLM Alloy

**Figure 10:**
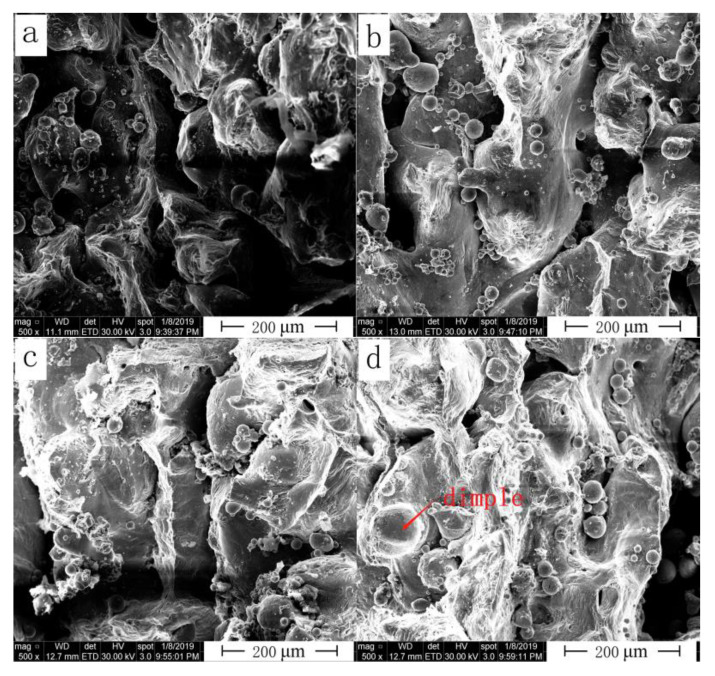
Fracture morphology of the SLM alloy formed at different laser powers (**a**) 380 W, (**b**) 400 W, (**c**) 440 W and (**d**) 460 W.

Surface and microtensile fracture morphologies of the SLM alloy were analyzed by field-emission environmental SEM (FEI Quanta450, FEI, Hillsboro, OR, USA). Variation of fracture morphology of the as-built samples formed under four different laser powers with a scanning speed of 900 mm/s and hatching distance of 0.06 mm are shown in Figure 10. When laser power was low, the fracture section of the alloy showed numerous unmelted powder particles accompanied by a large number of pores. High porosity of the material significantly decreased the range of material deformation. Due to limited deformation (strain value), the strength (ultimate strength) was also reduced. With increasing laser power, the fracture section started to show the characteristics of ductile fracture, small dimples were observed in Figure 10c,d, and the strength was evidently improved.

**Figure 11:**
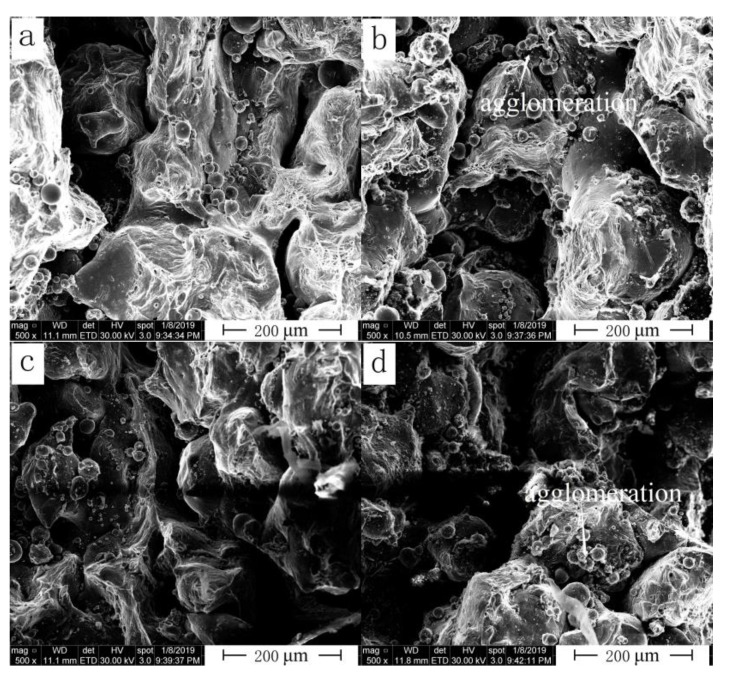
Fracture morphology of the SLM alloy formed at different scanning speeds: (**a**) 700 mm/s, (**b**) 800 mm/s, (**c**) 900 mm/s and (**d**) 1000 mm/s.

Variation of fracture morphology of the SLM alloy with increasing scanning speed for group *D* is shown in Figure 11. Fracture morphology shows the opposite trend to that of relative density. The higher the scanning speed, the lower the energy absorbed by the alloy powder, resulting in numerous unmelted powder particles. When scanning speed was high, some powder particles would agglomerate. These agglomerations resulted in discontinuous melting channels on the alloy surface, thereby forming a large number of micron-sized holes. These pores greatly reduced density and tensile strength of the SLM alloy. Increasing the density of the SLM alloy was therefore an important means to obtain SLM parts with good comprehensive performance.

**Figure 12:**
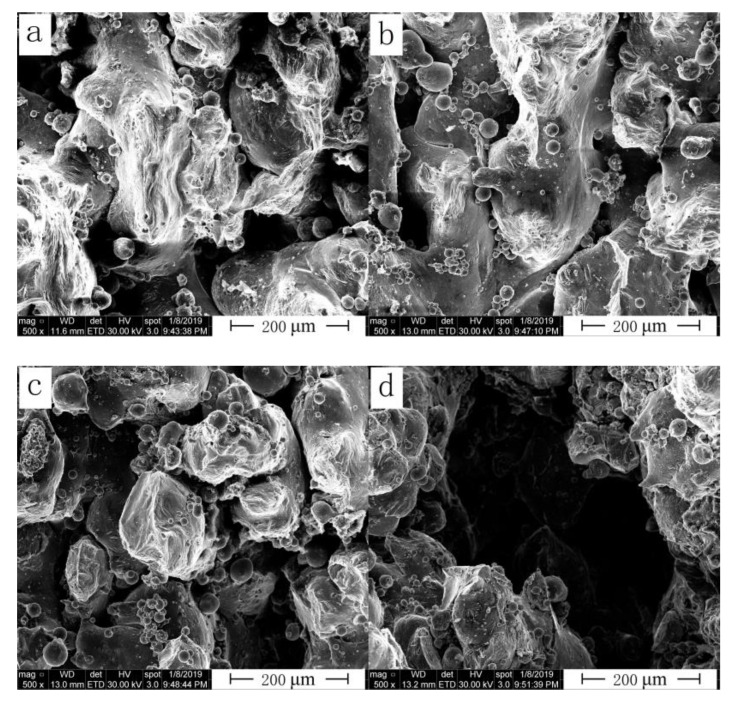
Fracture morphology of the SLM alloy formed at different hatching distances: (**a**) 0.05 mm, (**b**) 0.06 mm, (**c**) 0.07 mm and (**d**) 0.08 mm.

Variation of fracture morphology of the as-built SLM samples with hatching distance for group *C* is shown in Figure 12. A large hatching distance was used, and some areas between the two melting channels remained unmelted, resulting in pores or cavities, which greatly reduced alloy performance i.e., tensile strength, hardness and density of the SLM alloy. When hatching distance was small, a large number of overlapping areas existed between the melting channels, resulting in a rough surface of the alloy, and the overlapping of layers eventually reduced the comprehensive performance of the alloy. This result was consistent with the effect of hatching distance on the relative density and hardness of the SLM alloy.

### 3.7. Effect of Process Parameters on Surface Roughness of the SLM Alloy

**Figure 13:**
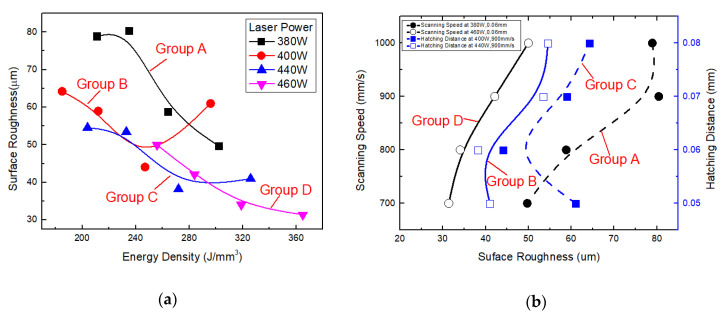
Surface roughness of the alloy formed by SLM under different process parameters: (**a**) laser power and surface roughness versus energy density of the alloy and (**b**) scanning speed and hatching distance versus surface roughness of the alloy.

Alloy powders are completely melted during the SLM process by a laser, and subsequently undergo rapid solidification [29,30]. Therefore, this process can be used to produce objects of high geometrical complexity. Variation of surface roughness of the SLM alloy with laser power, scanning speed and hatching distance for groups A, B, C and D (where a line corresponds to a group) was plotted in Figure 13. The results for groups A, B, C and D are shown in Figure 13a. As shown in the Figure 13a, surface roughness of the alloy decreased with increasing laser power. When laser power was 460 W, scanning speed was 700 mm/s, hatching distance was 0.06 mm and surface roughness of the SLM alloy reached the minimum value of 31.384 μm. Effect of scanning speed on surface roughness for groups *A* and *D* and that of the hatching distance on hardness for groups *B* and *C* are shown in Figure 13b. As shown in the figure, surface roughness of the alloy increased with increasing scanning speed for groups A and D. For group D, when scanning speed was increased from 700 to 1000 mm/s, surface roughness of the SLM alloy increased from 31.384 to 49.972 μm. Moreover, surface roughness increased when hatching distance was excessively low or high. This result was consistent with the effect of hatching distance on relative density of the SLM alloy.

**Figure 14:**
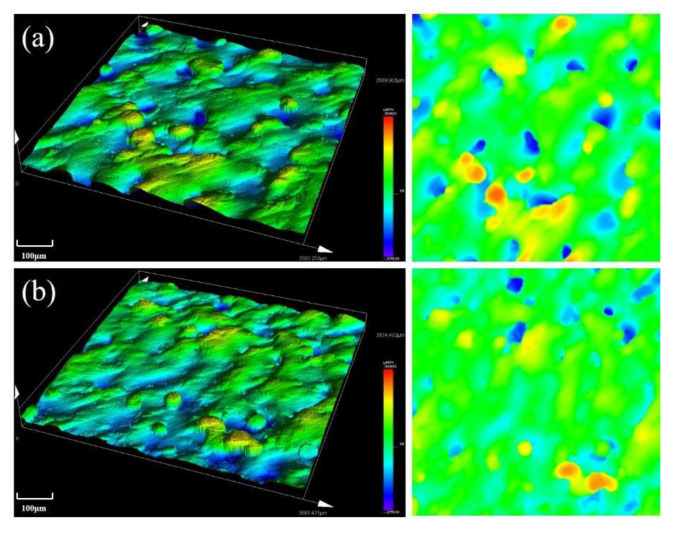
Surface topography of the SLM alloy formed at different laser powers: (**a**) 380 W and (**b**) 460 W.

Variation of surface topography of the as-built samples formed under two different laser powers with a scanning speed of 900 mm/s and hatching distance of 0.06 mm are shown in Figure 14. Depth and width of the molten pool were increased with increasing laser power, which reduced the spheroidization rate and improved the surface quality of the formed alloy. As shown in the figure, surface roughness of the alloy was rougher when laser power was 380 W.

**Figure 15:**
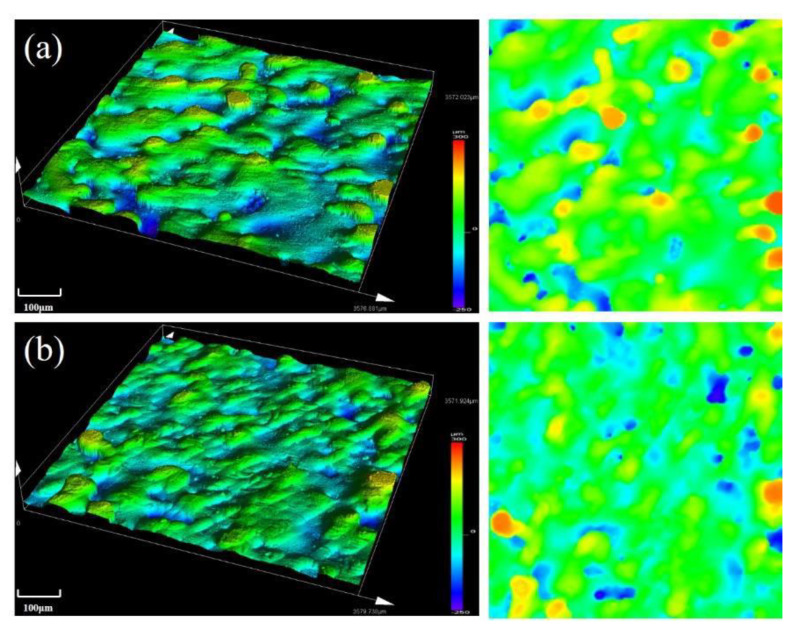
Surface topography of the SLM alloy formed at different scanning speeds: (**a**) 1000 mm/s and (**b**) 700 mm/s.

Variation of surface topography of the as-built samples formed under two different scanning speeds with laser power of 380 W and a hatching distance of 0.06 mm are shown in Figure 15. As shown in the figure, surface roughness of the alloy was rougher when scanning speed was 1000 mm/s. More alloy powder was not fully melted with increasing scanning speed, which worsened the surface quality of the formed alloy.

**Figure 16:**
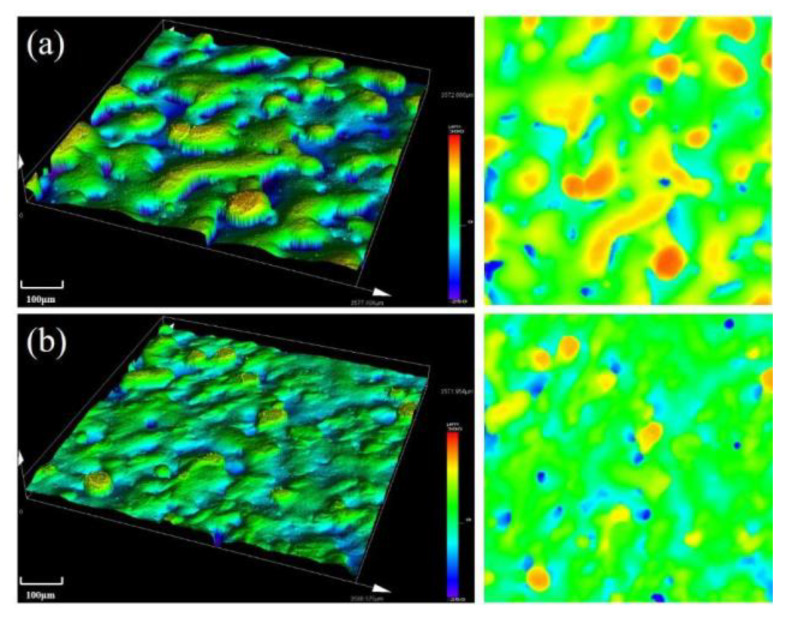
Surface topography of the SLM alloy formed at different hatching distances: (**a**) 0.08 mm and (**b**) 0.06 mm.

Variation of surface topography of the as-built samples formed under two different hatching distances with a laser power of 440 W and scanning speed of 900 mm/s are shown in Figure 16. As shown in the figure, surface roughness of the alloy was rougher when hatching distance was 0.06 mm. As scanning distance decreased, the overlapping ratio was also increased, so strong metallurgical bonding between the powder particles was achieved. However, smaller scanning distance caused excessive heat concentration of the laser beam, which produced deformation of the formed alloy and worsened the surface quality of the SLM alloy.

## 4. Conclusions

The range of process parameters for preparing the copper alloy powder by SLM was roughly determined by ANSYS simulation, and the Cu–Cr–Zr alloy with good comprehensive properties was fabricated by optimizing the influence of process parameters (laser power, scanning speed and hatching distance) on relative density, hardness, tensile strength and surface roughness of the SLM alloy. The main conclusions were drawn as follows:

(1) Relative density of the alloy increased with increasing laser power at the same scanning speed and hatching distance. The influence of scanning speed on the density was greater than that of hatching distance when the power was the same. When the laser power was 460 W, scanning speed was less than 1000 mm/s, the hatching speed was kept constant at 0.06 mm and high relative densities (>90%) were achieved compared to other processing parameters used for other groups.
(2) Hardness of the SLM alloy initially increased and then decreased with decreasing scanning speed and hatching distance. The possible reason is that when scanning speed is larger than inflection point speed, and the hatching distance is greater than inflection point distance, Cr atoms are mainly precipitated, which improves the hardness; otherwise, Cr atoms are mainly solid solution, which reduces the hardness.
(3) A high laser power resulted in strong metallurgical bonding of the SLM alloy and high tensile strength. High scanning speed resulted in low relative density and low tensile strength of the SLM alloy. Tensile strength initially increased and then decreased with increasing hatching distance.
(4) Alloy powders were completely melted during the SLM process by a laser, and subsequently underwent rapid solidification. Therefore, this process could be used to produce objects of high geometrical complexity. Surface roughness of the alloy was decreased with increasing laser power. When laser power was 460 W, scanning speed was 700 mm/s, hatching distance was 0.06 mm and surface roughness of the SLM alloy reached the minimum value of 31.384 μm.

To sum up, by increasing laser power and decreasing scanning speed, the comprehensive properties of the SLM alloy, i.e., tensile strength, were gradually improved. With increasing hatching distance, the comprehensive properties of the SLM alloy were initially increased and then decreased. The best comprehensive performance was obtained when laser power was 460 W, scanning speed was 700 mm/s and hatching distance was 0.06 mm. Highest tensile strength, hardness, roughness and density of the SLM alloy formed under those process parameters were 153.5 MPa, 119 HV, 31.384 μm and 91.62%, respectively.

## Figures and Tables

**Table 1:** Process parameters used in selective laser melting (SLM).

Group	Id	Laser Power (W)	Scanning Speed (mm/s)	Hatching Distance (mm)	Energy Density (J/mm^3^)
*A*	1	380	700	0.06	302
	2	380	800	0.06	264
	3	380	900	0.06	235
	4	380	1000	0.06	211
*B*	5	400	900	0.05	296
	6	400	900	0.06	247
	7	400	900	0.07	212
	8	400	900	0.08	185
*C*	9	440	900	0.05	326
	10	440	900	0.06	272
	11	440	900	0.07	233
	12	440	900	0.08	204
*D*	13	460	700	0.06	365
	14	460	800	0.06	319
	15	460	900	0.06	284
	16	460	1000	0.06	256
